# Endothelial cells during craniofacial development: Populating and patterning the head

**DOI:** 10.3389/fbioe.2022.962040

**Published:** 2022-08-29

**Authors:** Hiba Asrar, Abigail S. Tucker

**Affiliations:** Centre for Craniofacial and Regenerative Biology, Faculty of Dentistry, Oral and Craniofacial Sciences, Guy’s Hospital, Kings College London, London, United Kingdom

**Keywords:** angiogenesis, vasculogenesis, cell signalling, vascular biology, tooth, gland, neural crest

## Abstract

Major organs and tissues require close association with the vasculature during development and for later function. Blood vessels are essential for efficient gas exchange and for providing metabolic sustenance to individual cells, with endothelial cells forming the basic unit of this complex vascular framework. Recent research has revealed novel roles for endothelial cells in mediating tissue morphogenesis and differentiation during development, providing an instructive role to shape the tissues as they form. This highlights the importance of providing a vasculature when constructing tissues and organs for tissue engineering. Studies in various organ systems have identified important signalling pathways crucial for regulating the cross talk between endothelial cells and their environment. This review will focus on the origin and migration of craniofacial endothelial cells and how these cells influence the development of craniofacial tissues. For this we will look at research on the interaction with the cranial neural crest, and individual organs such as the salivary glands, teeth, and jaw. Additionally, we will investigate the methods used to understand and manipulate endothelial networks during the development of craniofacial tissues, highlighting recent advances in this area.

## Introduction

The vasculature acts as a major transport mechanism with blood as a constant flowing medium being distributed through vessels. It serves as a multipurpose delivery system providing essential nutrients and removing toxic metabolites (Rajendran et al., 2013). Blood vessels are part of a complex circulatory system responsible for development and maintenance of organ systems. Any dysregulation in the mechanisms underlying blood vessel regulation or abnormal blood vessel formation contributes to development of widespread pathologies including peripheral vascular diseases, metastatic conditions and bone diseases (Rajendran et al., 2013; Ramasamy et al., 2015).

Blood vessels formation is a highly organized sequential event comprised of two distinct mechanisms, vasculogenesis and angiogenesis, which occur throughout the body. These tightly regulated processes commence during embryonic life and continue postnatally. During embryonic development vasculogenesis begins in tissues through the *in-situ* differentiation of endothelial precursors forming an immature vascular network that coalesce to form *de novo* tubes (Schmeisser and Strasser, 2002). Post-natal blood vessel formation generally occurs through angiogenesis, involving new vessels sprouting from pre-existing vessels (Moore, 2002). The entire vasculature is lined chiefly by endothelial cells. These cells carry out primary roles as cell barriers controlling movement of cells and substances in and out of blood vessels. Moreover, they control critical functions such as regulation of vascular tone, coagulation and homeostasis (Sandoo et al., 2010). A number of proteins, such as CD31 (also known as PECAM1) is present evenly over the cell surface and can be used as an endothelial cell marker. In embryonic tissues endothelial cells keep pace with growth and development, whereas in adult tissue they continue to allow renewal, remodelling and reconstruction. During early vascular development important molecular signals such as Bone morphogenetic protein-4 (BMP4) initiate endothelial cell differentiation from various multipotent mesenchymal cells while fibroblast growth factor (FGF) stimulates cells by inducing early endothelial markers (Dejana et al., 2017). Vascular endothelial growth factor (VEGF) expression controls blood vessel growth and remodelling, providing mitogenic and survival stimuli to endothelial cells, with FGF signalling in endothelial cells controlling the sensitivity of the response (Murakami and Simons, 2008). Endothelial cells acquire support from contractile cells called pericytes and smooth muscle cells (collectively referred to as mural cells) that attach to the abluminal surface of endothelial cells (Armulik et al., 2011). These mural cells/perivascular cells express common markers, such as neuron- glial-antigen (NG2), Cd146, α-SMA, and platelet derived growth factor- β (Bergers and Song, 2005).

The focus of this review is on the function of endothelial cells (ECs) in the craniofacial region and the molecular signatures that regulate endothelial cell migration in the cranium. We discuss perfusion independent roles of the vasculature in directing tissue morphogenesis/cell differentiation in different cranial structures. Moreover, we discuss technical advancements that aid in the investigation of the roles of endothelial cells.

## Endothelial cell origin in the cranial region

**FIGURE 1:**
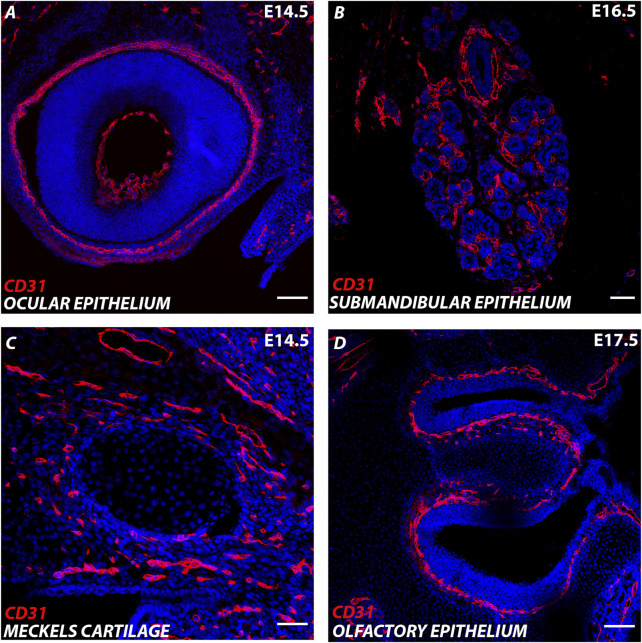
Endothelial cells in the developing cranial region. **(A–D)** Murine cranial tissue CD31 expression in red. Nuclei stained with DAPI in blue. **(A)** E (embryonic day) 14.5 Developing eye. **(B)** E16.5 Developing submandibular gland. **(C)** E14.5 Developing Meckel’s cartilage (centre) surrounded by CD31 cells in the surrounding mesenchyme. **(D)** E17.5 Developing olfactory epithelium. Scale bar = 100 μm.

The craniofacial region houses the sense organs, brain and masticatory organs, all of which are well supplied by the vasculature (Figure 1). The face is created from the fusion of the pharyngeal arches and nasal processes (lateral, medial and frontal) that form during early craniofacial development. Endothelial cells develop around the forming pharyngeal arch arteries in the centre of each pharyngeal arch and are derived from *Mesp1*-lineage positive mesoderm (Liang et al., 2014). In contrast, the surrounding pericytes and smooth muscle cells are derived from the neural crest (Etchevers et al., 2001). Previously, tracing of the embryonic vasculature suggested that the vasculature of the pharyngeal arches formed as an extension of the dorsal aorta, with the endothelial cells sprouting from the larger vessel (angiogenesis) (Hiruma et al., 2002). However, genetic labelling using *Tie2cre* has revealed that vessels in the arches arise *de novo* directly from the pharyngeal mesoderm (vasculogenesis), rather than as extensions of existing vessels (Li et al., 2012).

Two distinct sources of endothelial cells have been described to populate the pharyngeal arches (Wang et al., 2017). The exact source of embryonic tissue that gives rise to anterior arch endothelium in mice is still unclear, but in the more posterior pharyngeal arches (arch 3–6) the endothelial cells derive from the secondary heart field (SHF) (Wang et al., 2017). The SHF is a subset of the *Mesp1* progenitor population defined by the *Isl1* transcription factor (Verzi et al., 2005; Evans et al., 2010). Quantitative analysis revealed that 95% of the endothelial cell population in pharyngeal arch 3–6 was derived from the SHF. VEGFR2 expressing cells in the SHF delaminate and migrate into the pharyngeal mesenchyme to form a primitive vascular network (Wang et al., 2017). These small blood vessels undergo remodelling to create the pharyngeal arch arteries in arch 3–6th, which eventually give rise to the aortic arch arteries (Hutson and Kirby, 2007). Even after extensive vascular remodelling the aortic arch arteries retain the SHF derived endothelium (Wang et al., 2017).

Vasculogenesis of the pharyngeal arch arteries is regulated by retinoic acid signalling (Li et al., 2012). Compromised retinoic acid signalling caused defects in both the pharyngeal endoderm and arch arteries, with retinoic acid receptor activity required for proper coalescence of endothelial cells into nascent blood vessels in the pharyngeal mesoderm (Wendling et al., 2000). *Rara1/Rarb* mutants showed bilateral absence or hypoplasia of the 4/6th arch artery (Li et al., 2012). *Mesp1cre/Rara* conditional knock out embryos had no effect on terminal differentiation of endothelial cells but led to scattered endothelial cells that failed to aggregate to form nascent vessels, highlighting the need for retinoic acid signalling for effective vasculogenesis (Li et al., 2012).

In zebrafish, time lapse imaging using the transgenic *Etsrp:GFP* line, where GFP labels vascular endothelial and myeloid progenitors, identified two bilateral angioblast clusters called the rostral organizing centre and midbrain organizing centre, which gave rise to cranial vessels (Proulx et al., 2010). These organizing centres contained endothelial clusters that formed by vasculogenesis and eventually gave rise to cranial vessels through angiogenesis (Proulx et al., 2010). At the 14–15 somite stage angiogenic extensions from the rostral organizing centre developed and endothelial cells migrated posteriorly and laterally giving rise to most rostral cranial vessels (Proulx et al., 2010). In contrast, the midbrain organizing centre progenitors collectively migrated and formed midbrain/hindbrain cranial vessels.

*Etsrp* is a Ets transcription factor that controls vascular and haematopoietic development and is homologous to *Etv2* in mammals. In *Etsrp* morphant zebrafish, the cranial vasculature was absent and scattered angioblasts were observed (Proulx et al., 2010). Interestingly, the endothelial progenitors appeared to change fate in the absence of *Etsrp*, forming skeletal muscle, suggesting a role for this transcription factor in determining endothelial fate (Chestnut et al., 2020).

## Migrating with the cranial neural crest

The cranial vasculature shares common migration pathways with cranial neural crest cells (McKinney et al., 2016). Neural crest cells form important structures in the craniofacial region, which are intimately linked with the cranial vasculature. Alteration in neural crest cell migration can give rise to craniofacial syndromes, known as neurocristopathies (Trainor, 2010). Cranial neural crest cell and endothelial cells, despite having distinct origins, migrate towards a common region in close proximity (McKinney et al., 2016). The vasculature is regulated by a family of essential growth factors called Angiopoietins (Davis et al., 1996; Suri et al., 1996) that are also highly expressed in cranial neural crest cells, the otic vesicle and neural tube (McKinney et al., 2016). During migration, frequent collisions were recorded between endothelial and r6 neural crest cells that led to changes in endothelial cell behaviour and cell shape (McKinney et al., 2016). Overexpression of *Angiopoietin 2* in neural crest cells perturbed endothelial cell migration and motility, with fewer, more unstable endothelial sprouts that underwent regression (McKinney et al., 2016). The interaction appears to go both ways, as highlighted in conditional *Flk1 (VEGFR2)* mutants. *VEGFR2* is indispensable for development of endothelial and haematopoietic lineages, with knockdown of this gene resulting in embryonic lethality during early embryogenesis due to disruption of the endothelial pool in the anterior region of the developing head (Eichmann et al., 1997; Shibuya, 2011). In the absence of cardio-cranial endothelial cells, the cranial neural crest cells were also deficient, with loss of neural crest markers in the second pharyngeal arch (Milgrom-Hoffman et al., 2014). Interestingly, loss of endothelial cells led to changes in the extra cellular matrix, which then impacted neural crest survival and migration and led to cell death of the cranial neural crest cells (Milgrom-Hoffman et al., 2014). This research, therefore, suggests an instructive role of the cranial vasculature in controlling neural crest decisions.

Analysis of *Ephrin* mutants, however, suggests that the cues that control the guidance of the cranial neural crest and vasculature are complex. In the *Ephrin B2* null mouse, both the cranial neural crest and the vasculature were defective, with an absent or significantly reduced second branchial arch (hyoid) and loss of the associated blood vessels (Adams et al., 2001). Endothelial cells were irregularly arranged and failed to form tubular network with numerous abnormal sprouts and branches invading somitic tissues (Adams et al., 2001). In contrast, loss of the cytoplasmic domain of *Ephrin B2* in a truncated mutant caused vascular and angiogenic defects in the head but did not disrupt cranial neural crest cell migration (Adams et al., 2001). These findings suggest the importance of full length *Ephrin B2* for remodelling and formation of cranial vasculature. However, a conditional knockin mouse, where *Ephrin B2* was rescued in the vasculature in *Ephrin B2* null mice, but not in the neural crest, led to normal neural crest migration. The defect in the neural crest in the mutant is, therefore, due to the defect in the vasculature (Lewis et al., 2015). These findings highlight the importance of endothelial-neural crest cell coordination during early stages of head morphogenesis.

## Guiding endothelial cells in the head

Endothelial cells need to be guided to particular organs during development and form an integral part of their development. This involves key molecules that guide endothelial cells throughout the body by a combination of attraction and repulsion.

The VEGF signalling pathway has been shown to be an important vascular attractant in a range of different tissues (Shibuya, 2011). VEGFR2 is largely found in high calibre arteries whereas VEGFR3 expression is restricted to veins and capillaries (Rocha and Adams, 2009). In the developing head, the vasculature expresses *VEGFR2* (Shadad et al., 2019). VEGF expression has been followed using LacZ reporters throughout mouse embryogenesis, with high levels in the cranial region, particularly associated with the developing tooth, whisker follicles, pituitary, and choroid plexus at E14.5 (Miquerol et al., 1999). In the whisker follicle, VEGF was expressed in the condensing mesenchyme but not in the epithelium, while in the tooth, VEGF was expressed in the dental epithelium, not in the dental papilla but in the mesenchyme of the dental sac (Miquerol et al., 1999). Expression of *VEGF* in the tooth epithelium is maintained throughout development and is closely associated with the enamel knots, signalling centres that control tooth morphogenesis (Shadad et al., 2019). *VEGF* has additionally been shown to be expressed in the mesenchyme surrounding Meckel’s cartilage (Wiszniak et al., 2015), a transient structure that forms the template for the lower jaw in vertebrates (Svandova et al., 2020). Craniofacial organs, therefore, have an organ specific expression pattern of VEGF, attracting the VEGFR2 positive vasculature to different regions of the cranial organs as they develop.

Sema3 family members compete with VEGF to bind to Nrp1 to inhibit VEGF induced angiogenesis (Parker et al., 2012). Several *in vitro* studies have explored the role of Sema3s in controlling endothelial cell migration (Gu and Giraudo, 2013). Chick forelimb studies reveal how Sema-3A coated beads disintegrate vascular network assembly whereas exogenous application of Sema-3A antibody led to local rescue of vasculature (Bates et al., 2003). During craniofacial development, semaphorin class 3 members such as *Sema3A Sema3B Sema3C* and *Sema3E* are expressed in developing brain stem, otic vesicle, eye (periocular mesenchyme), trigeminal ganglion and branchial arches (Chilton and Guthrie, 2003). In the first pharyngeal arch, *Sema 3C* was expressed mainly in the arch epithelium, while *Sema3D* was expressed in a patch in the arch mesenchyme (Chilton and Guthrie, 2003). Studies on murine embryonic development have reported expression of *Sema3G* in the retinal/brain endothelial cells and *Sema3A/Sema3C* in the submandibular gland (Chung et al., 2007; Tan et al., 2019; Chen et al., 2021). Similarly, zebrafish studies have reported expression domains of *Sema3G* in the midbrain, diencephalon, telencephalon and pharyngeal arches during early development. In the retina, *Sema3G* is expressed in endothelial cells and acts as a vascular remodelling factor. Conditional deletion of *Sema3G* in endothelial cells led to hyper-pruned vascular networks with leaky immature retinal vessels (Chen et al., 2021). Semas, may therefore, have a greater role in remodelling the vasculature, rather than in guiding migration as first envisioned. VEGF, thus, guide the endothelial cells into the head to reach specific organs at distinct timepoints, and then Semas act to remodel the vasculature.

## Instructive role of endothelial cells in controlling morphogenesis/cell differentiation in craniofacial structures

As observed for the neural crest, the vasculature not only functions as a transport system bringing nutrients and oxygen and removing waste but can have an instructive role controlling cell migration and building organ architecture. These perfusion independent roles have been studied in the forming head in the context of the salivary glands, tooth, jaw cartilage and brain.

### Endothelial cell control of salivary gland morphogenesis

**FIGURE 2:**
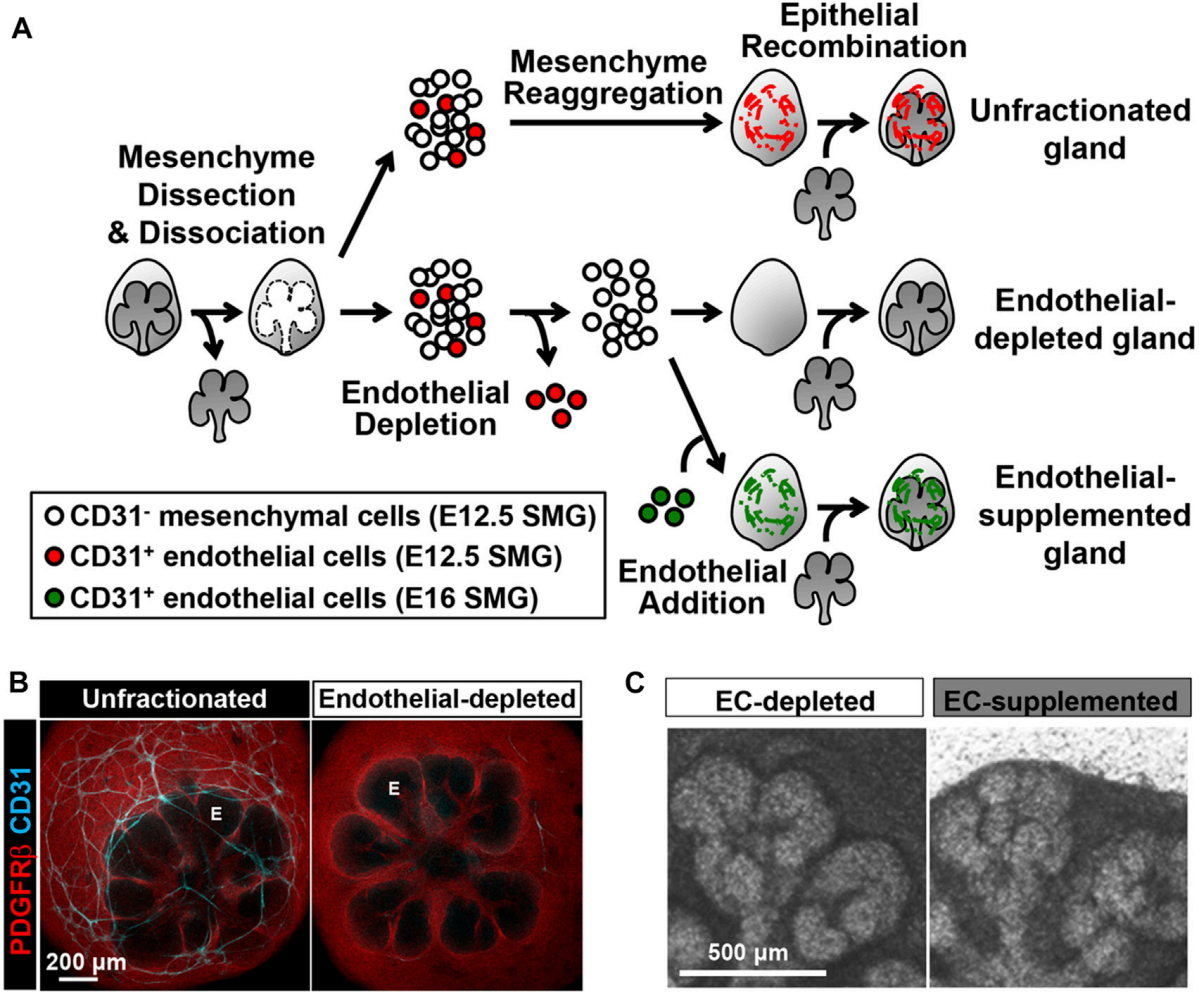
CD31 cell-dependent vasculature development promotes epithelial patterning in an SMG cell fractionation/reconstitution assay. **(A)** SMG cell fractionation/reconstitution assay schematic. Unfractionated SMG mesenchyme amenable to cell immunodepletion was generated by microdissection of the mesenchyme from the epithelium followed by enzymatic dissociation of the mesenchyme to single cells and re-aggregation of the isolated mesenchymal cell population. Re-aggregated mesenchyme was then reconstituted with an intact microdissected E13 epithelial rudiment. For endothelial cell depletion, CD31^+^ endothelial cells were immunodepleted from fully dissociated mesenchyme cells using MACS with CD31 microbeads prior to re-aggregation of the dissociated mesenchyme and reconstitution with an intact epithelium. For endothelial cell supplementation, endothelial-depleted mesenchymal cells were mixed with MACSisolated endothelial cells collected from E16 glands, prior to re-aggregation of the mesenchyme and reconstitution with an intact epithelium. The reconstituted glands were cultured *ex vivo* for 48 h post-reconstitution **(B)** Confocal images (maximum projection images) consistently showed a change in the epithelial patterning (no marker, black) with a mesenchymal marker (PDGFRβ in red) defining the mesenchymal shape. CD31^+^ vasculature (cyan) was present in unfractionated, but not in endothelial depleted mesenchyme. E, endbud. **(C)** Endothelial supplementation promoted epithelial branching. Taken with permission from Kwon et al., 2017.

During development, CD31+ve endothelial cells intimately surround developing salivary glands wrapping around the epithelial end buds from E12.5 onwards (Kwon et al., 2017). Notably, VEGFR2+ cells were detected inside clefts that define the boundary between terminal proacinar structures and secondary duct epithelium. Blocking VEGFR2 tyrosine kinase activity with inhibitors such as ZM323881 and SU5416 or with VEGFR2 siRNA, inhibited vascular development in culture and altered epithelial branching with fewer endbuds, wider excretory ducts and decreased total ductal area (Kwon et al., 2017). These findings suggest that the vasculature is essential for submandibular gland morphogenesis. Immuno-depletion of CD31^+^ endothelial cells, using a novel *ex-vivo* SMG cell fractionation/reconstitution assay, led to loss of the vasculature and increased end bud size (Kwon et al., 2017) (Figure 2). Epithelial gland architecture was rescued by supplementation of the depleted gland cultures with endothelial cells, which supported end bud formation with thin secondary ducts and partially restored the vasculature (Kwon et al., 2017). Inhibition of VEGFR2 resulted in longer and wider K19 + developing ducts with expansion of K19+ ductal population and reduction of Kit+ cells relative to the control group. Interestingly, exogenous addition of endothelial cell-regulated mesenchymal factors such as IGFBP2 and IGFBP3 restored epithelial patterning (Kwon et al., 2017). These results highlight that endothelial cells can promote expansion of progenitor populations and suppress premature ductal differentiation during SMG development.

### Angiocrine factors facilitating jaw and cartilage morphogenesis

During embryonic development, VEGF is strongly expressed in the neural crest derived mesenchyme surrounding Meckel’s cartilage (Wiszniak et al., 2015). Genetic ablation of *VEGF* from cranial neural crest cells led to cranial hypoplasia, cleft palate, maxillary bone defects and an abnormal bow shaped mandible (Wiszniak et al., 2015). In these conditional mutants, neural crest cell specification and migration into the first pharyngeal arch was not altered excluding the possibility of VEGF directly influencing early NCC development (Wiszniak et al., 2015). *VEGF* deletion perturbed chondrogenesis and led to the formation of a dysmorphic Meckel’s cartilage which failed to acquire its normal arrowhead morphology. Persistence of this abnormal phenotype between E14.5 to E17.5 confirmed defective growth of the cartilage linked to a defect in vascularisation of the first arch (Wiszniak et al., 2015). Downregulated CD31 expression confirmed reduction of micro-vessel density and loss of mandibular artery in mutant jaws. *In vitro* co-culture of arterial tissue with neural crest cell or chondrocytes confirmed that neural crest cell derived VEGF regulates blood vessels, which in turn secrete angiocrine factors to instruct chondrogenesis. A similar defect in the mandibular artery resulting in craniofacial defects was observed in the *Tie2creNrp1fl/fl* mouse, validating the instructive role of blood vessels during jaw morphogenesis (Wiszniak et al., 2015).

As an extension to this study, *invitro*/*ex-vivo* tissue explants were used to investigate specific vascular mitogens that might promote Meckel’s cartilage proliferation to facilitate jaw extension. A murine chondrogenic cell line, ATDC5, was stimulated with aorta-conditioned media, which activated insulin receptor *Akt,ERK1/2* and *Stat3* (Marchant et al., 2020). RT-PCR revealed high expression of *IGF1*, minimal expression of *IGF2*, and no detectable levels of *Ins1* or *Ins2*, highlighting that IGF1 is the main angiocrine factor secreted from aortic rings (Marchant et al., 2020). In keeping with this, IGF1 was reduced in cranial neural crest specific conditional *VEGF* mutants, suggesting that the cranial blood vessels are a key source of IGF1 driving mandibular extension (Marchant et al., 2020). Genetic deletion of *IGF1* from endothelial cells resulted in a shortened Meckel’s cartilage with reduced proliferative capacity while exogenous supplementation of IGF1 rescued the proliferation deficit in cultured mandibles. These findings point towards a crucial angiocrine function of IGF-1 during craniofacial cartilage development.

### Angiogenic-odontogenic coupling during tooth development

*VEGF* is highly expressed in the developing tooth bud (Miquerol et al., 1999) and all endothelial cells in the surrounding vasculature show high immunoreactivity for *VEGFR2* (Shadad et al., 2019). Postnatally, endothelial cells at the periphery of the tooth express *VEGFR1* and *VEGFR2* concomitantly at high levels (Matsubara et al., 2022). These *Vegfr + ve* capillaries lacked smooth muscle coverage, expanded and perforated the basal layer of odontoblasts (dentin producing cells) before the onset of active dentinogenesis (Matsubara et al., 2022). Deletion of *VEGFR2* from endothelial cells postnatally led to loss of the vasculature by apoptosis, particularly near the odontoblast layer, and a knockon effect on the width of the odontoblasts and dentin delayed odontoblast maturation. Metabolomic analysis of *VEGFR2* null mice revealed a decrease in ATP/ADP and creatine phosphate levels, indicating a decline in the phosphate pathway, highlighting a potential systemic effect on the teeth. Interestingly, the peripheral endothelial cells were shown to express *TGFβ1, Ptn* and *Jag2*, all of which are known to promote odontoblast maturation, with addition of these factors leading to rescue of dentin markers in the conditional *VEGFR2* knockout in culture (Matsubara et al., 2022). These results highlight the importance of the vasculature in facilitating post-natal tooth development and maintaining dentin mineralization by the provision of multiple angiocrine factors.

### Bidirectional neural/endothelial communication regulating oligodendrogenesis

TGFβ1 produced by endothelial cells has additionally been shown to impact development of oligodendrocyte progenitor cells (OPCs) (Paredes et al., 2021). During embryogenesis, CNS vascularization coincides with neural progenitor cell proliferation and differentiation (Peguera et al., 2021). Notably, blood vessels develop intimately aligned to neural progenitors without migrating into neurogenic regions (Nie et al., 2010). *Angiopoeitin-1* (*Ang1*), produced by the CNS, was shown to signal through *Tie2* on endothelial cells, which in turn signalled back to the OPCs to regulate their specification (Paredes et al., 2021). *TGFβ1* expression in endothelial cells coincided with OPC specification (Hamaguchi et al., 2019). Endothelial cell derived TGFβ1 was shown to act downstream of *Ang1/Tie2* with reduced pSMAD3+Olig2+ progenitors in *Ang1 fl/fl* ^*Nestin:Cre*^ embryos, highlighting the endothelial-neural bidirectional cross talk necessary for coordinated development (Paredes et al., 2021). Notably, recombinant TGFβ1 was able to rescue neural specification in spinal cord explants from *Ang1fl/fl* ^*Nestin:Cre*^ and *Tie2 fl/fl* ^*Pdgfb:CreERT2*^ transgenic mice.

An understanding of the instructive role of the vasculature during development in multiple organs, highlights the importance of providing endothelial cells when constructing tissues and organs. It also highlights the issue with many explant culture techniques, where the vasculature is not maintained over time, resulting in loss of potentially key signals *in vitro*.

## Technical advances to study the role of endothelial cells

In order to understand the instructive signals provided by cranial endothelial cells further, various hurdles need to be overcome. We need to be able to identify different subpopulations of endothelial cells to understand tissue specific roles. We need to be able to isolate tissue-specific endothelial cells to investigate the effects of depletion and augmentation *in vivo* and *in vitro*. We need to understand the cross talk between cranial organs and endothelial cells, and be able to track endothelial cells *in vivo* to understand dynamic tissue relationships. Luckily new culture techniques, new biomaterials, imaging techniques, sorting techniques, transcriptomics, and the use of transgenic animals, all make these areas a reality.

### Identifying endothelial subpopulations

Endothelial cells can be identified by cell markers such as CD31, CD34, endo-mucin and ib4 (Goncharov et al., 2017). However, endothelial cells exhibit heterogeneity and express unique transcriptional signatures with different properties depending on their location (McCarron et al., 2019). The level of Notch activation, for example, can regulate their proliferation state (Chesnais et al., 2022). With this in mind it is important to understand tissue specific roles and contexts for endothelial cells. An excellent understanding of endothelial cell heterogeneity during different developmental timepoints and in different developing tissues can be achieved through single cell RNA sequencing. Using this method, a unique endothelial subtype was identified in the tooth specialised for dentinogenesis (Matsubara et al., 2022). Interestingly, in the cardiac field, developing endothelial cells initially segregated by lineage but later by tissue localisation, with adult endothelial cells being more homogeneous with respect to lineage and location (Phansalkar et al., 2021). Drastic changes in retinal endothelial gene expression were also observed during postnatal development, between P6 and P10 (Zarkada et al., 2021). These changes were attributed to EC maturation, arterial specification, proliferation and blood-retina barrier formation (Zarkada et al., 2021). scRNA-seq analysis identified two distinct cell clusters (D-Tip and S-Tip) guiding retinal vascularisation and expressing known tip cell markers such as *Mcam, Chst1, Nid2* and *Rhoc*. Several markers were identified to differentiate between both tip cell types, however D-Tip cells displayed higher ECM genes but lower TCA cycle and glycolysis scores as compared to S-Tip cells, indicating different ECM and metabolic need (Zarkada et al., 2021). Notably, D-Tip cells displayed higher TGFb signalling scores and genetic ablation of *TGFb receptor* using *Cdh5Cre* driver led to haemorrhagic malformations (Zarkada et al., 2021). These findings have helped in the identification of unique transcriptional signatures of D-Tip retinal endothelial cells that acquire blood retina barrier characteristics for proper retinal vascularization (Zarkada et al., 2021). Therefore, sc-RNA seq can help identify different endothelial clusters and their functions in various organs during development.

### Sorting and recombining endothelial cells

Given the above heterogeneity of endothelial cells during development, it is important to be able to isolate organ-specific endothelial cells in order to investigate tissue patterning. Fluorescent activated cell sorting (FACS) offers flexibility and allows separation of multiple cell population, however, to isolate specific cells from large populations magnetic activated cell sorting (MACS) has been shown to be more advantageous (Liao et al., 2016). Kwon et al. (2017) utilized MACS and gland reconstitution techniques to investigate perfusion independent roles of endothelial cells during salivary gland morphogenesis (Figure 2). For isolation of CD31+ve endothelial cells, primary mesenchymal cells from glands at key stages were suspended with mouse CD31 microbeads for immunomagnetic separation (Kwon et al., 2017). This technique allowed stage and tissue specific endothelial cells to be isolated in order to investigate the impact of endothelial cells on gland architecture (Kwon et al., 2017).

Tissues can be deconstructed and reconstituted with and without endothelial cells, or with additional sources of endothelial cells added (Figure 2). Such reconstitution assays are an excellent experimental technique to study perfusion independent effect of endothelial cells and have been successfully carried out to understand the role of endothelial cells during salivary gland development (Kwon et al., 2017). Additionally, these reconstitution techniques can be adapted to combine endothelial cells from different tissues and different stages in order to understand how stage of differentiation and tissue specific signatures impact the vascular-tissue crosstalk (Figure 2).

In addition to reconstituted explants, organoids provide an excellent way to understanding cell behaviour and recapitulating development taking a reductionist approach. Organoids created from organ-specific cells can be cultured with and without endothelial cells to answer questions about morphogenesis and differentiation. This method has been used to study the impact of endothelial cells on mammary gland organoids, where the culture system allowed epithelial branching to recapitulate many aspects of mammary gland development (Wang et al., 2021).

### Imaging cranial endothelial cells *in vivo* and in culture

**FIGURE 3:**
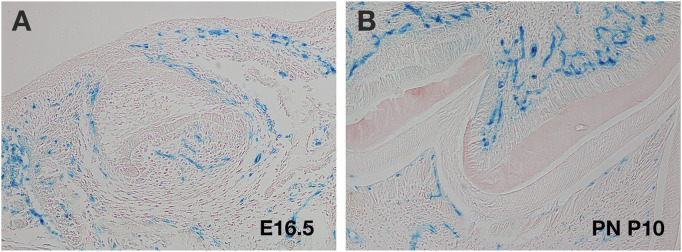
Use of *Mesp1cre* transgenics to follow cranial endothelial cells. **(A,B)** Murine molar tooth in *Mesp1creLacZ* mouse (blue cells). **(A)** E16.5 late cap stage showing migration of endothelial cells into the papilla. **(B)** Postnatal (PN) day 10 M tooth showing invasion of endothelial cells on both sides of the dental hard tissue.

Several transgenic mice have been utilised to label endothelial cell populations during different developmental stages (Payne et al., 2018). In the neural crest derived parts of the head (which include the facial region and front of the calvaria), the only mesodermal components are the muscles and endothelial cells (Yoshida et al., 2008). *Mesp1cre* reporter mice, which label the mesoderm, are therefore particularly useful to follow the development of the cranial vasculature. These mice have been used to study the contribution of endothelial cells to developing cranial tissue over time, with tooth germ explant cultures used to follow migration of endothelial cells into the dental papilla (Rothova et al., 2011) (Figure 3). Additionally, *Vegfr* reporter lines, have been used to follow the contribution of endothelial cells to different tissues, allowing high resolution of endothelial cells structure to visualise structures such as filopodia (Matsubara et al., 2022). Such labelling techniques, combined with the ability to image at high resolution with light sheet microscopy, means we can now trace endothelial cell movement, and follow the process of migration and remodelling *in situ* in 3 and 4D (Prahst et al., 2020).

### Making vascular scaffolds to enhance development and repair

Several attempts have been made to tissue engineer vascular scaffolds to allow angiogenesis and vasculogenesis *in vitro* and *invitro* studies. Techniques such as lyophilization, electrospinning, decellularization and 3D bioprinting are frequently used in vascular tissue engineering. To incorporate beneficial properties of natural materials researchers have fabricated scaffolds with fibronectin, fibrin, elastin, Matrigel, collagen loaded with cells and reported vascular tissue formation (Cooper and Sefton, 2011; Yang et al., 2020). Collagen1/fibronectin gel implants seeded with endothelial cells have facilitated capillary formation and vascular network assembly in mice (Cooper and Sefton, 2011). Some cell types, such as adipose stromal cells have been shown to release signals to encourage angiogenesis (Rehman et al., 2004). However, in the presence of a nanostructured collagen-based scaffold the release of angiogenic factors, such as VEGF, was reduced in human adipose-derived stem cells as they underwent differentiation (Borgese et al., 2020). There is therefore a need to create scaffolds that enhance not hamper angiogenesis.

To overcome degradation problems associated with natural polymers, several synthetic materials such as hydrogel based polymers, poly (acrylonitrile-co-methyl-acrylate (PAN-MA) polyglycolic acid (PGA), poly-L-lactic acid (PLLA), polyhydroxyalkanoates (PHA’s) and poly ethylene glycol (PEG) have been used in scaffold fabrication for tissue vascularization (Wang et al., 2022).

Scaffolds that encourage angiogenesis have been investigated in the context of dental stem cells. Dental stem cells have been extensively researched due to their ease of accessibility and the availability of multiple sources in the oral cavity providing a promising source of cells for bone and dental regeneration (Cappare et al., 2020). Co-cultures of periodontal ligament stem cells and umbilical cord endothelial cells have been used to prevascularise scaffolds to promote osteogenic differentiation (Zhao et al., 2021). Such scaffolds can then be combined with other techniques to encourage osteogenesis, such as the use of low-level laser irradiation (Ballini et al., 2015). Prevascularisation can reduce the time-period when implanted scaffolds are avascular, and thereby reduce the time when cells are subject to hypoxic conditions. However, whether such prevascularised scaffolds using non-tissue specific endothelial cells can provide the correct instructive signals as highlighted above, is yet to be determined. Interestingly, dental pulp stem cells (DPSCs) can also be induced to differentiate into endothelial cells, by culture in gel moulds (Sasaki et al., 2020). These DPSC endothelial cells can then support pulp-like tissue regeneration (Katata et al., 2021). DPSCs are neural crest derived while endothelial cells are mesodermal, emphasising the plasticity of the neural crest in being able to form a wide variety of tissue types. These studies highlight the benefits and issues associated with tissue engineered vascular scaffolds, but overall, such scaffolds have great potential to lead to novel regenerative therapeutic solutions.

## Conclusion

In this review, we have discussed the origin of cranial endothelial cells and highlighted key signalling molecules and other cell types that influence their migration into the craniofacial region. Once the endothelial cells have arrived, they not only provide a source of nutrients and waste removal, but actively contribute to the development of cranial organs by provision of angiocrine factors. In the head these signals have been identified so far as IGFs and Tgfbs. Tgfb1 has been shown to play a key instructive role in both the tooth and the brain, highlighting that some of these signals may not be tissue specific but have general functions in the control of differentiation. Instructions from the vasculature are also required for tissue engineering strategies to encourage repair and regeneration of cranial tissue. In keeping with this need, new tissue engineering strategies are being developed to encourage vascularisation of cranial tissue, either through host tissue recruitment or pre-vascularisation of engineered tissue (Li et al., 2022). For enhanced repair and to simulate regeneration, there is much to be learn from understanding the expanding role of endothelial cells in cranial development.
